# Practical Notes

**Published:** 1891-02

**Authors:** 


### Practical Notes.

### BRONCHIAL ASTHMA.

℞ Iodide of ammonium, dr. ij.
Fl. ext grindelia robusta, ℥ ss.
Fl. ext. glycyrrhiza, dr. iv.
Tinct. lobelia,
Tinct. belladonna, aa dr. ij.
Syr. tolu, q. s. ad. ℥ iv.

Dose: Teaspoonful three times a day; extra doses during a paroxysm.

This formula may be varied to suit indication. I have cured many cases of asthma by means of it, some of over twenty years' standing, myself among the number.—*Dr. Covert, in Am. Med. Jour.*

### GONORRHŒA.

BY DR. J. W. WHITE, PHILADELPHIA.

Dr. J. W. White recommends the use of the following mixture, in capsules, for treatment of acute urethritis. In about two-thirds of his cases the discharge ceased within a week. In the majority it was necessary also to use an injection, and for this he recommends 2 to 10 grains of sulpho-carbolate of zinc in a 10 to 15 per cent. solution of peroxide of hydrogen:

R Salol, 3 1-2 grains.
Oleoresin of cubebs, 5 grains.
Balsam of copaiba (Para,) 10 grains.
Pepsin, 1 grain.—*Ex.*

---

Dr. Hirst gives an equivalent of human milk:

℞ Cream, f ʒ iv.

Cow's milk, f ʒ ij.

Water, f ℥ j.

Milk sugar, gr 1.

Sterilize by steaming for twenty minutes, and before using add lime water, f ʒ ij.—*Ex.*

Dr. Hare gives for acute stage of bronchitis in children :

℞ Tr. aconiti, gtt. xij.
Syr. ipecac, f ℥ ss–j.
Liq. potassii citratis, q. s. ad. f ℥ iij.

M. and S. One teaspoonful every three hours.

For the later stages :

R Ammonii chloridi, ʒ j.

Ext glycyrrhizæ fl., f ʒ iv.

Aquæ dest. q. s., ad. f ℥iij.

M. and S. One teaspoonful three times a day.—*Ex.*

### TREATMENT OF FISSURED NIPPLE.

BY DR. ELOY, FRANCE.

(*a*) *Preventive Treatment :*

1. Extreme cleanliness—after each nursing we should carefully wash the parts.
2. Apply a three per cent. lotion of boric acid.
3. If the areola is sensitive, use the following pomade :

R Carbonate of zinc, gr. lxxv.

Glycerine, ʒj.

Vaseline, ʒvj.,

Or also, the following :

R Tannin, ʒj. gr. xlv.

Glycerine, ʒ xijss.

Rose water, ʒv.

(*b*) *Curative Treatment :*

1. Cauterization of the fissure with nitrate of silver stick.
2. Immediately afterwards use the following :

R Collodium, ʒ v.
Ether, gtt. xlv.
Hydrochlorate of cocaine, gr. 3-10.

Or :

R Salol, gr. xxx.
Ether, gtt. lx.
Hydrochlorate of cocaine, gr. ij 1-4.
Collodium, ʒ vj, gr. xv.

---*Leonard's Ill. Med. Jour.*

### LA GRIPPE.

℞ Phenacetin, grs. 20.
Sul. morphia, gr. i.
Quiniae Sulph., gr. 30.
Capsici, grs. 10.
M. ft. Caps. No. 10. One every 3 hours.

J. S. TODD.

℞ Quinia sulph., grs. xij.
Pulv. dov. com., grs. vj.
Hydrg. chlo. mit., gr. ij.
Ft. cap 2. Take every 4 hours for 24 hours. For intercostal neuralgia, large mustard plaster over chest.

G. G. ROY.

---

TETANY IN CHILDREN.—Dr. J. Lewis Smith, whom the world recognizes as the Nestor of Pediatrics, believes in the treatment of the tetany of children with the bromide of potassium. It is one of the most useful remedies. He commends four grains dissolved in cold water every three or four hours for a child from one and a half to two years. It is an entirely safe remedy and usually causes a diminution or cessation of the spasms. He condemns the Indian hemp, chloral and hypodermic injections of morphine which have been employed in adult cases.—*Medical Mirror.*

---

TREATMENT OF SOFT CHANCRES.—Drop peroxide of hydrogen solution upon the surface until the pus is removed and bubbling ceases. Bathing with warm water before making the application is advisable, This can be done morning and evening, and a simple water dressing applied upon absorbent cotton, or the black wash used where a stimulating effect is desired. If a dry dressing is preferred, the following formula:

℞ Hydrarg. chlor. mitis, ʒ j.

Bismuth. sub-nitrat.,

Pulv. cinchonæ flavae, aa ʒ ij. M.

has proved very efficient and is recommended by Dr. I. G. Davis, of Bridgeton, N. J.—*Dietetic Gazette.*

### A PROCEDURE FOR WASHING OUT THE MALE BLADDER WITHOUT THE USE OF A CATHETER.

---

Dr. Rotter describes, in the *Centralblatt fur die Gesammte Therapie*, July, 1890, a method for washing out the male urinary bladder without the use of the catheter, by which the introduction of infectious germs into the bladder by the catheter is thus avoided. The author employs a rubber tube attached to an irrigator filled with lukewarm antispeptic fluid, on the end of which is a mouth-piece, wrapped with antiseptic gauze and covered with vaseline, to render its insertion into the orifice of the urethra more readily performed. The tube is filled with liquid, and air allowed to escape, and then inserted for about half an inch within the urethra. Immediately before the operation the patient must first empty the bladder, and is then placed upon his back, with the legs separated and flexed upon the pelvis, the hips being somewhat raised; the end of the tube is inserted into the urethra, and held fast with the fingers, and the fluid allowed to enter. Within one or two minutes, it is stated, the sphincter of the bladder relaxes and, after three minutes, the liquid enters the bladder.

The pressure may be regulated by the height to which the irrigator reservoir is elevated. After the removal of the tube, the patient may readily empty his bladder of the fluid introduced, which may amount to a pint or more.—*Therapeutic Gazette. International Journal of Surgery.*

---

A NOTE ON THE MANAGEMENT OF CONSTIPATION IN INFANCY.—In a recent clinic, Dr. Woodbury, of the Medico-Chirurgical College of Philadelphia, mentioned a very useful plan of treating constipation in early childhood. He said that the temptation is great in these cases to give calomel or mercury with chalk, on account of the smallness of the dose and the ease of administration owing to the tastelessness of the remedy.

Unless there was also a history of congenital syphilis, or some constitutional reason for the administration of mercurials, he did not consider it judicious to prescribe them simply for their laxative effect. For an emergency, however, where there is constipation with irritability of the stomach, he would give a quarter of a grain of calomel with two grains of saccharated pepsin every hour or two until the bowels were evacuated. For Simple constipation, where the child passes hard masses of fœces and has a screaming fit every time the bowels are moved, he now instructs the mother to give Carlsbad water, in tablespoonful doses, four or five times a day. The child takes it as readily as plain water, and it has the most happy effect; partly from the quantity of water, but principally from the increase of secretions all along the intestinal tract, including the liver, caused by the special action of this well-known water.—*Dietetic Gazette*

---

ACUTE TONSILLITIS; PHAGEDENIC CHANCROID.—A male, aged 22, of good physique, was admitted to the venereal wards of Philadelphia Hospital suffering with chancroids, which had destroyed the greater part of the prepuce and attacked the glands. He was put to bed and the following lotion applied:—

℞ Ferri et potassæ tartratis, - - gr. x.
Aquæ, - - - - - - f℥j.
M. Fiat sol.
Sig.: Wash parts every three hours.

Internally:—

℞. Ferri et potassæ tartratis, - - ʒij.

Syrup. zingiberis, - - - - f℥j.

Aquæ bullientis, - - - - f℥v.

M. Fiat sol.

Sig.; fʒij t. d.

Under this treatmeant he rapidly improved. About the sixth day after admission he complained of tenderness and swelling in his throat. Upon examination he was found to be suffering with acute tonsilitis. His temperature was 105.2, with severe rigors. The pus was at once evacuated, and he made a good recovery in a few days. The tinct. guaiac. ammon. was given early as abortifacient without success, though it gave marked relief later. Dose, fʒj in milk as a gargle.—S. T. B., in *The Times and Register.*

THREE DOUBLE BIRTHS.—The wife of John Bean of Valley Falls, aged 65 years, gave birth to twins on Monday evening. Her daughter, Mrs. Stratton, who lives in a neighboring township, presented her husband with twins the same evening. Mrs. Stratton's daughter Eva was married a year ago and lives in Arrington. The friends of Mrs. Stratton and her mother were not yet through congratulating them over the interesting natal coincidence in their families when Mrs. Stratton received a letter from her son-in-law that her daughter had given birth to twins herself on Mondary evening. The three double births occurred within ten minutes of each other.—*Kansas City Med. Journal.*

---

TREATMENT OF RICKETS.—The treatment of rickets should be by food rather than by drugs. Raw meat is of more value than iron, and cream or fresh milk than cod liver oil. The diet must be carefully examined to see that it contains a due portion of fat, proteids and salts. A sufficiently close estimate is easily made since the composition of milk and of all food used for children is accurately known. The amount of animal fat in a rickety child's food must equal at least one-fourth of the total solids taken; proteids and carbo-hydrates about one-third, and salts about one-tenth. Such a diet will cure rickets without drugs. Iron is often a useful adjunct. The salts of lime may be added in the form of lacto-phosphate. Potent aids are sunlight, fresh air and warm clothing.—*Indiana Medical Journal.*

---

KING'S JOURNAL DIRECTORY for 1891, containing a complete list of Medical, Dental, Pharmaceutical, Chemical, Microscopical, Sanitary, Veterinary and Medico-Legal Journals, both Home and Foreign, will be ready for delivery on or before Jan. 1st next. Orders should be sent promptly as the book is sold by subscription only. Price, 50c. post-paid. Address Dr. F. King, Publishers, Box 587, New York.

---

Ten drops tincture of iodine in a small goblet of water will usually arrest the nausea and vomiting that succeed an over- indulgence in strong liquor.

INFLAMMATION OF BREAST SIMULATING CANCER.—Dr. G. Phocas *Gazette des Hopitaux*, August 19, 1890, describes a form of diffuse inflammation of the breast which has been observed by himself and by several distinguished Parisian surgeons. It occurs about the menopause. Its cause may be chronic irritation from clothing, overwork, etc. A tumor forms, independent of lactation, and occupies the greater part of the glandular tissue of the breast. It is bulky, often hemispherical, firm and uniform in consistence. Its surface is irregular. From its periphery proceed prolongation of the gland involved in the disease just as in cancer. In two cases at least the tumor spread into the nipple. The skin is often free, but sometimes adherent. The tumor has never been found to adhere to the pectoralis major. The patient complains of pricking sensations and slight pain in the breast. The tumor is usually tender to pressure. Most important is it to bear in mind that the axillary glands may be enlarged. A characteristic of this form of inflammation of the breast is its irregular course. Instead of increasing steadily, whether slowly or rapidly, it sometimes reaches a great size in a few days, and then slowly diminishes in volume, or else it grows smaller all of a sudden, and increases again after a long time, taking a long while to resume its former bulk. The resemblance to cancer is very great, and the tumor mostly looks like an advanced scirrhus. It diminishes in volume after a few weeks' treatment. Dr. Phocas, Professor Verneuil and others prefer free pulverizations with carbolic acid.—*Supp. Brit. Med. Journal.*

---

CARBOLIC ACID ANTIDOTE.—It appears to be not generally known that soluble sulphates completely antidote either carbolic acid or creasote, no matter how given, for when they meet they form a harmless compound (sulpho-carbolic acid).

---

### TO OUR ADVERTISERS.

When advertisers desire to change their matter they will please forward the copy for such change so that it will reach us prior to the 20th of the month preceding month of issue.

---

### SOME NOTES BEARING ON THE ADMINISTRATION OF IRON.

BY JOHN AULDE, M. D., PHILLADELPHIA, PA.

Although iron is highly esteemed as a medicament, and is largely used for its tonic effect upon the system, so frequently does it occur that the patient objects, owing to some idiosyncrasy or fancy, that we cannot regard it wholly as an ideal hæmatinic. No apology, therefore, is required in offering to the profession a comparatively recent preparation, which is free from some of the objections that have been urged against many of the iron preparations now in use. In order to make the reasons which I have to offer clear and distinct to the casual reader, I have deemed it wise to consider briefly some points intimately connected with the pharmacology of the drug. From this preliminary study we shall be in a measure prepared to estimate how nearly the new product comes to meeting the defects with which we have had to contend so long, and at the same time it may possibly lead to a more intelligent use of this well-known remedy.

Besides the reduced iron, we have in general use the ferric and ferrous preparations, the latter being more mild, less astringent, and ree from the objections to the ferric salts—that of coagulating albumin. Lethal doses of the ferric salts used intravenously, in experimental investigations, cause almost immediate paralysis of the central nervous system, fall of blood-pressure, and death. Although the perchloride, when thus used, causes instant death by coagulation of the blood, it does not act in this direct manner when introduced subcutaneously; the nerves are unaffected, but at the points of elimination inflammatory action is set up, *e, g.*, the kidneys, liver, and intestinal mucous membrane show more or less effect.

Absorption takes place as a peptonate or albuminate, but it is taken up so slowly that no appreciable result follows, unless, as just stated, it may be used intravenously or subcutaneously. Absorption takes place more rapidly in catarrhal conditions of the intestinal tract—a fact to be borne in mind when exhibiting large doses, which cause gastro-intestinal catarrh. Small doses do not have this effect, nor does the metal appear in the urine from their administration, such as may be observed after the ingestion of large doses. It will be inferred from the foregoing that by the exhibition of small doses of a soluble preparation of iron it will be assimilated without causing derangement of the alimentary tract, and in this way the secondary effects, *i*, *e*., the deposit of the metal in the system, may be avoided.

The fact should be kept constantly in view, that metals have a poisonous action upon nerves, nerve centres, muscles, and upon all glandular structures; and as iron is a reputed hæmatinic, much harm may result from its injudicious employment, as there are evidently certain toxic effects following the long-continued use of insoluble preparations. This is a rule which applies especially to all insoluble iron preparations, and it is but reasonable to assume that, whatever harm has been done through this means, may have escaped attention, because few physicians are likely to envestigate the presence of facttiious diseases. Another factor which has contributed to lessen these evils, is the slow process of absorption.

The foregoing observations apply with equal force to the effects of the drug upon the circulatory apparatus. While copper is an active agent in causing contraction of the blood-vessels, iron produces slow contraction, showing that it is less irritant (stimulant) to the nervous system. This may possibly be accounted for on the hypothesis that iron is a normal constitutent of the blood. Whether this effect is due to irritation (stimulation) of the vaso-motor nerves, central or peripheral, or to a direct action upon the muscular walls of the blood-vessels, is a question still in doubt. My own impression is that through the influence of the medicament upon the nerve-cells the large doses, comparatively, arrest their function, when contraction of the muscular structures in the vessels takes place. The ferric salts, owing to their property of coagulating albumin and blood, of course produce more marked effects than the ferrous salts. Digitalis and ergot among the organic, remedies, well-known as vascular tonics, furnish apt illustrations and barium-chloride among the inorganic of this important principle.

Iron has a tendency to accumulate in the liver; small doses do not show this tendency, but they may serve to increase the functional activity of this organ, when given in a soluble, non-astringent form, by restoring cell-nutrition to the normal.

The effect of iron upon muscular structure has long been known to experimental physiologists, but I doubt if this knowledge is appreciated by many practitioners, who regard the possible benefits to be derived from the exhibition of iron preparations in proportion to the amount tolerated by the patient. Now, large doses, while they do not affect the irritability of muscular structure, lessen materially the amount of work it is capable of performing, while small doses increase the capacity of muscle for work. What is most to be desired, therefore, is a preparation not open to the objections inferred from these investigations; but owing to the necessity for consulting the palate of our patients, it is also desirable that the substance should be free from the nauseating effects which are so common to all preparations of iron. The combination, I believe, is to be found in that form known as levulose ferride, which was highly recommended to me several years ago by my friend, Dr. James Collins, of this city.

The preparation known as levulose ferride is one which takes the place of a well-known and popular German product, called *Eisenzucker* (iron sugar), very extensively used in domestic practice. I was led to the employment of iron-sugar on account of its palatability, fastidious patients and children making no objections to it; but this has been supplanted by levulose ferride, which in the form of tablet triturates will be taken as readily as chocolate bon-bons. It is readily soluble in an excess of water, and practically free from any ferruginous taste or styptic effect when dissolved in the mouth, and is substantially a peptonate. The method of preparing it is briefly as follows: To a certain amount of iron a measured quantity of malt-sugar (maltose) is added, and the mixture constantly stirred while exposed on a water-bath. While it possesses all the desirable qualities mentioned, the presence of metallic iron may be determined by chemical analysis, the strength of the product being about three per cent.

This preparation, it will be apparent, will act much less actively as an astringent than even the ferrous preparations; but, of course, it cannot be expected to take the place of the ferric products, which are sometimes demanded, as in the case of intestinal parasites (sarcina ventriculi and lumbricoides). On the other hand it will be especially indicated for the relief of anæmia and chlorosis, owing to its ready absorption, lack of astringency, and its palatability. In all cases of defective nutrition, from any cause, where the ingestion of any form of medicament is a trial to the patient, this product will be kindly received. A synopsis of some of the cases in which it is indicated, together with a summary of the effects following its employment, may prove interesting to the physician.

During the early summer months, I had under observation a young mother with a six-months old child, who presented a very anæmic condition. I had seen her but once since the delivery of her child, and anticipating that she would not be able to nourish it sufficiently and maintain her health, I had cautioned her in regard to the most appropiate diet. Notwithstanding every care had been used, she was finally compelled to seek medical aid, or go to bed. All that this patient required was something for the purpose of increasing the amount of hæmoglobin, which would restore the integrity of the red corpuscles and improve the oxygen-carrying capacity of the blood. This being most readily accomplished by levulose ferride, she was ordered to take tablets of this preparation, each containing three grains, after meals. To meet the emergency, and increase the patient's strength until such time as the advantages of the iron would be apparent, small doses of strychnine (one-sixtieth grain) were administered along with the iron. Ordinarily, this class of patients, when they begin in the early summer, suffer more or less from the effects of the heat, and become regular patrons of the doctor; but this patient did not make her appearance again for about two months, when she said she thought it was about time to have a little more of the same medicine. I may mention in passing, that the first medicine was sufficient only to cover the first ten days, and the patient seemed greatly disappointed that she was compelled to return.

So many children are so promptly benefited by the use of a small quantity of iron, that it is a great drawback to us that no palatable preparation has been discovered and put on the market. I have in mind a little fellow, who has long been very much adverse to eating meat, due, I presume, to defective digestion; but for the past few weeks, since he has been taking the levulose ferride, he seems quite content to eat meat alone, and is becoming strong and robust. Not long ago I had a visit from a lady, who brought with her a young lad, aged fourteen, who had a most forbidding cadaveric expression, and he could eat no meat. His brother, I was told, had died at about this age from Bright's disease, and this one presented all the symptoms peculiar to the brother who died. Still, with attention to diet, outdoor exercise in the country, and a tablet trituate containing three grains of levulose ferride after meals, he made a prompt recovery. Although I was unable to discover any symptoms of Bright's in this instance, I was impressed with the depression due to the anæmic condition; and yet, without some readily assimilable iron preparation, it would have been a tedious process to start him on the way toward recovery.

Late in the spring of the year, a gentleman, aged about thirty-five, called on me, complaining of dyspepsia, although he had been under the treatment of another physician for overwork for the preceding four years. After regulating his diet, and adopting treatment calculated to restore the activity of the digestive apparatus, he was placed upon levulose ferride along with strychnine sulphate—three grains of the former in tablet form, and one-sixtieth grain of the latter, and did remarkably well on this combination. This product, like all other mild preparations of iron, is mostly indicated in cases of this class, and along with these may be mentioned chorea, convalescence from lingering diseases, like typhoid fever; and in all such instances, I venture to anticipate that the results will be especially favorable where proper attention is given to dietetic measures.

The administration of the remedy may be confined to the use of the powder, which is taken dry on the tongue, dissolved in water or coffee; or it will be found more convenient in the form of tablets, each containing three or five grains. The dose for children ranges from three to ten grains, and for adults from five to thirty grains.

The Levulose Ferride was obtained through Messrs. Eisner & Mendelson Co., of New York, who import this article.

